# Commentary: Next Generation Sequencing and Linkage Analysis for the Molecular Diagnosis of a Novel Overlapping Syndrome Characterized by Hypertrophic Cardiomyopathy and Typical Electrical Instability of Brugada Syndrome

**DOI:** 10.3389/fphys.2017.01056

**Published:** 2017-12-12

**Authors:** Michelle M. Monasky, Giuseppe Ciconte, Luigi Anastasia, Carlo Pappone

**Affiliations:** ^1^Arrhythmology Department, IRCCS Policlinico San Donato, Milan, Italy; ^2^Stem Cells for Tissue Engineering Lab, IRCCS Policlinico San Donato, Milan, Italy; ^3^Department of Biomedical Sciences for Health, University of Milan, Milan, Italy

**Keywords:** Brugada syndrome, tropomyosin, myofilaments, hypertrophic cardiomyopathy, ajmaline

We read with great interest a recently published report by Mango et al. (2016) describing for the first time an association between hypertrophic cardiomyopathy (HCM), Brugada syndrome (BrS), and an α-tropomyosin (*TPM1*) gene missense mutation (c.574G>A; p.E192K) identified using a combined approach of integrated linkage analysis and next generation sequencing (NGS). Using NGS enables the analysis of a large number of genes quickly and simultaneously and at a reasonable cost, but the amount of information obtained is extremely large and difficult to interpret. Using integrated linkage analysis to narrow the focus of the NGS limited the amount of data obtained to an amount that could be more easily analyzed. Thus, the authors were able to quickly identify the tropomyosin mutation found in the family members affected with clinical characteristics of both HCM and BrS.

Other studies support the idea that arrythmogenic sudden death, even in the absence of overt structural abnormalities, can be directly linked to altered sarcomeric properties (Baudenbacher et al., 2008). HCM is associated with a high susceptibility to sudden death (Alves et al., 2014) and has long been linked to myofilament mutations (Yar et al., 2014; van der Velden et al., 2015). To investigate the functional effect of the E192K mutation in tropomyosin, Mango et al. investigated in silico the effect of the interaction between TPM1 and polycystin-2 protein (PKD2), which is an intracellular Ca^2+^ channel that interacts with different sarcomeric proteins regulating Ca^2+^ signaling (Mango et al., 2016). The modeling experiments suggested that the E192K mutation can result in significant changes in tropomyosin structure by disrupting the formation of the homodimeric coiled-coil quaternary structure, leading to disruption of the ability of TPM1 to interact with PKD2, potentially altering the function of PKD2 in regulating Ca^2+^ ion flux. The tropomyosin E192K mutation has never before been associated with BrS, and thus this is an interesting finding that provides an indication for future experiments that should spark much interest in the field, especially since BrS is commonly thought to originate as a channelopathy. Commonly, the explanation given for the ECG abnormalities observed in BrS is that BrS is a channelopathy characterized by ion channel dysfunction and that it has been linked to mutations in genes encoding for subunits of cardiac sodium, potassium, and Ca^2+^ channels, the most common mutation being SCN5A, which accounts for 15–30% of BrS cases (Kapplinger et al., 2010). However, although it is the most common mutation found in BrS, the SCN5A mutation is not found in the majority of BrS patients. Thus, no single causal factor appears to link all BrS patients (Amin et al., 2008). The results of Mango et al. present a novel and interesting finding that BrS may not always originate as ion channel dysfunction, but may also originate as myofilament pathologies that alter Ca^2+^ signaling. This provides an indication for future experiments to investigate sarcomeric alterations in BrS.

While the results presented by Mango et al. are thought-provoking and provide justification for future inquiries, caution should be taken in concluding that for certain an overlap between HCM and BrS is caused by the specific tropomyosin mutation described. The number of participants for the study is only seven, and only four of which exhibit clinical manifestations of HCM and BrS and carry the mutation. Importantly, all of the participants are from the same family. Thus, it is important to verify the findings in a larger patient population and in other families.

Flecainide or ajmaline can be used to provoke the type 1 BrS ECG pattern in patients to confirm the diagnosis of BrS. Mango et al. administered the flecainide test to diagnose BrS in the patients who carry the tropomyosin mutation. As future studies are performed to verify the results of Mango et al., it is important to note that flecainide is less effective than ajmaline in unveiling the type 1 BrS ECG pattern. In fact, in one study, only 68% of patients who tested positive with an ajmaline test were positive with the flecainide test (Wolpert et al., 2005). Therefore, it would be better to use ajmaline, rather than flecainide, to affirmatively diagnose BrS in future studies. The occurrence of false negatives could mislead researchers who attempt to verify the results of Mango et al. or who use this approach to diagnose new clinical entities.

In conclusion, caution should be taken in concluding that the α-tropomyosin gene missense mutation identified is for certain the cause of a novel overlapping clinical entity without confirmation in a larger sample size. Nevertheless, Mango et al. have for the first time identified a sarcomeric mutation associated with BrS, and this warrants further investigation and is of great interest to the field, suggesting that BrS may not always originate as ion channel dysfunction, but may also originate as myofilament pathologies that alter Ca^2+^ signaling.

## Author contributions

MM: wrote and edited the commentary; GC, LA, and CP: edited the commentary.

### Conflict of interest statement

The authors declare that the research was conducted in the absence of any commercial or financial relationships that could be construed as a potential conflict of interest.
